# Co-morbidity in patients with early rheumatoid arthritis - inflammation matters

**DOI:** 10.1186/s13075-016-0928-y

**Published:** 2016-01-28

**Authors:** Lena Innala, Clara Sjöberg, Bozena Möller, Lotta Ljung, Torgny Smedby, Anna Södergren, Staffan Magnusson, Solbritt Rantapää-Dahlqvist, Solveig Wållberg-Jonsson

**Affiliations:** Department of Public Health and Clinical Medicine, Rheumatology University Hospital, Umeå, 90185 Sweden; Department of Rheumatology, Sunderby Hospital, Luleå, 97180 Sweden; Department of Rheumatology, Östersund Hospital, Östersund, 83183 Sweden; Department of Rheumatology, Sundsvall Hospital, Sundsvall, 85186 Sweden

**Keywords:** Early rheumatoid arthritis, Co-morbidity, Inflammation

## Abstract

**Background:**

Patients with rheumatoid arthritis (RA) suffer from co-morbidities that contribute to a shortened lifespan. Inflammation is important for the development of cardiovascular disease, but little is known on its relationship with other co-morbidities. We investigated the role of inflammation for the development of new comorbidities in early RA.

**Methods:**

Since 1995, all patients with early RA in Northern Sweden are included in a prospective study on co-morbidities, with a total of 950 patients being included. At the time for this study, 726 had been ill for ≥5 years. Data on co-morbidities, clinical and laboratory disease activity and pharmacological therapy were collected from patient records and further validated using a questionnaire at RA onset (T0) and after 5 years (T5).

**Results:**

Of the patients, 53.2 % of the patients had one or more co-morbidity at onset, the commonest being: hypertension (27.3 %), obstructive pulmonary disease (13.9 %), diabetes (8.0 %), hypothyroidism (6.3 %) and malignancy (5.0 %). After 5 years, 41.0 % had developed at least one new co-morbidity, the most common being: hypertension (15.1 %), malignancy (7.6 %), stroke/transient ischemic accident (5.1 %), myocardial infarction (4.3 %) and osteoporosis (3.7 %). Age at disease onset, a raised erythrocyte sedimentation rate (ESR) at inclusion, previous treatment with glucocorticoids (GC; p < 0.001 for all), extra-articular RA (Ex-RA; p < 0.01), DAS28 (area under the curve) at 24 months (p < 0.05), previous smoking at inclusion (p = 0.058) and male gender (p < 0.01) were associated with a new co-morbidity overall at T5. Treatment with biologics (p < 0.05) reduced the risk. In multiple logistic regression modelling, ESR (p = 0.036) at inclusion was associated with a new co-morbidity after 5 years, adjusted for age, sex, smoking and GC treatment. In a similar model, Ex-RA (p < 0.05) was associated with a new co-morbidity at T5. In a third model, adjusted for age and sex, a new pulmonary co-morbidity was associated with a smoking history at inclusion (p < 0.01), but not with ESR.

**Conclusion:**

There was substantial co-morbidity among early RA patients already at disease onset, with considerable new co-morbidity being added during the first five years. Measures of disease activity were associated with the occurrence of a new co-morbidity indicating that the inflammation is of importance in this context.

## Background

Rheumatoid arthritis (RA) is a chronic inflammatory autoimmune disease with unknown etiology that primarily affects the peripheral joints and, over time, leads to loss of mobility if untreated. RA is also a systemic disease and many patients have constitutional symptoms (e.g., weight loss, fatigue, etc.) at disease onset. Some patients also develop extra-articular manifestations (Ex-RA), a known risk factor with prognostic implications [1, 2]. It is well-known that RA patients have increased mortality [3–5] and shortened life expectancy compared with the general population [3, 6, 7]. In clinical practice, it is not unusual that at their first visit to a rheumatology clinic patients with early RA already have one or more co-existing diseases [8–11], with additional increased risk of work-related disability, impaired quality of life and substantial societal costs [12]. Some of the co-existent diseases are causally associated with RA, others occur as a consequence of the pharmacological treatment of RA [10, 12, 13]. Co-morbid conditions that are over-represented in RA patients compared with the general population include cardiovascular disease (CVD) [3, 14–17], interstitial lung disease [18–20], infections [21, 22], gastrointestinal disease [12, 23] and osteoporosis [24, 25]. There are no convincing data for evidence of greater risk of malignancy, although a slight increase in overall risk has been reported [26]. However, there is a trend towards certain site-specific cancers, such as lymphoma [26, 27] and lung cancer [26, 28], being more common in patients with RA. It has been shown that morbidity [16, 29] and premature mortality [3, 4, 30] due to CVD is increased in RA patients in comparison to population rates. Several studies during the past decade have demonstrated a link between inflammation and the development of CVD in patients with RA [31–33]. However, little is known about the importance of the inflammatory status in relation to other co-morbidities in patients with RA.

In the present observational study, an inception cohort of patients with early RA has been followed prospectively from disease onset. The aims were: first, to investigate the presence of co-morbidities at onset of RA in all patients who had been included; second, to investigate the development of new co-morbidities in those who had been ill for >5 years at follow up, during the first 5 years following diagnosis of RA; and finally, to assess the role of inflammation in relation to those new co-morbidities.

## Methods

By reference to the nation-wide Swedish Early RA Register, part of the Swedish Rheumatology Quality Register [34], all eligible patients from the four northernmost counties of Sweden diagnosed with early RA (i.e., symptomatic for <12 months), and fulfilling the American Rheumatism Association classification criteria [35] were consecutively included from December 1995 in a large observational study on the progression of RA, including development of co-morbidities. By November 2012, 950 patients (649 women, 301 men) registered with newly diagnosed early RA had been included in the study at the time of being diagnosed with RA (T0 baseline/inclusion). By November 2012, 224 patients had been ill for <5 years and thus, only baseline data have been registered for these patients. In all, 726 patients had been ill for 5 years or more and could be evaluated in the 5-year follow-up (T5). Of these 726 patients, 52 died before they reached 5-year follow up. No patient was lost to follow up. All patients were clinically examined regularly by their local rheumatologist, and routine laboratory tests and radiographs of hands and feet were performed.

According to the study protocol for inclusion into the Early RA register, the 28-joint count of tender joints (TJC) and swollen joints (SJC), a visual analog scale (VAS) for pain and the patient’s global assessment, completion of the Health Assessment Questionnaire (HAQ) [36] and measurement of inflammatory markers, i.e., erythrocyte sedimentation rate (ESR) and C-reactive protein (CRP) were recorded at baseline and after 6, 12, 18, 24, 36, and 60 months. These data were downloaded for the purpose of this study. The accumulated disease activity score in 28 joints (DAS28) was calculated only up to 24 months [37] because data were incomplete from some centers after 24 months.

The presence of autoantibodies, i.e., rheumatoid factor (RF), and anti-nuclear antibodies (ANA), was assessed at baseline by routine laboratory methods in current use at each of the participating hospitals. Antibodies against cyclic citrullinated peptides/proteins (ACPA) were analyzed at baseline using enzyme-linked immunoassay (ELISA) for anti-CCP2 antibodies (Euro-Diagnostica). Genotyping for PTPN22 1858C/T polymorphisms and HLA–DRB1 (shared epitope; 0101/0401/0404/0405/0408) typing were performed as previously described [38, 39].

All patient records were carefully examined and data collected according to the study protocol, on co-morbidities and pharmacological treatment, both at inclusion (T0) and after 5 years (T5). The patients also completed a self-reported questionnaire on co-morbidity at T0 and T5 to further increase the validity of the collected data. The questionnaire comprised specific questions about CVD and an open question about other previous and current co-existing disorders. All co-morbidity data have been processed in accordance with the classification of diseases described by Charlson [40]. The basis for the Charlson instrument is a disorder sub-division consisting of 10 somatic disease categories (myocardial, vascular, pulmonary, neurologic, endocrine, renal, liver, gastrointestinal, cancer/immune and miscellaneous), each with several sub-groups [40]. Recorded myocardial and vascular variables were myocardial infarction (MI)/coronary artery bypass graft (CABG), stroke/transient ischemic attack (TIA)/deep vein thrombosis (DVT)/pulmonary embolism (PE), and ruptured aortic aneurysm. The procedure and criteria for data collection of cardiovascular co-morbidity has previously been described in detail [41].

For all other diagnoses, including malignancy and neurologic, renal, liver and gastrointestinal disease, a diagnosis recorded by a clinician was accepted as a sufficient basis for registration. The presence of hypertension was further defined as receiving anti-hypertensive treatment. A diagnosis of pulmonary disease, including asthma and chronic obstructive pulmonary disease (COPD), required continuous or intermittent bronchodilator treatment. Endocrine disease was defined as the presence of diabetes mellitus, osteoporosis, hyperparathyroidism, hyperthyroidism, goitre and hypothyroidism. Treatment with thyroxine was required for a diagnosis of hypothyreosis. The diagnoses were further validated by information in the patients self-reported questionnaires. A disease mentioned only in the self-reported questionnaire that could not be validated in the records was not registered. To distinguish extra-articular RA, including RA-associated lung disease from co-morbidities, the Malmo criteria for RA-associated lung disease were used [42]. Cumulated pharmacological treatment was registered (months before inclusion and during the follow up period) on use of corticosteroids and disease-modifying anti-rheumatic drugs ((DMARDs), i.e., methotrexate, sulfasalazine, chloroquine, azathioprine, mycophenolatmophetil, myocrisine, auranofin, cyclosporine, leflunomid, alkylating cytotoxic agents) including biological agents (etanercept, adalimumab, infliximab, anakinra, rituximab). Treatment with non-steroidal anti-inflammatory drugs (NSAIDs) before inclusion and any period during the follow up period, was recorded simply as “yes” or “no”. The regional Ethics Committee at the University Hospital of Umeå approved this study and all participants gave their written informed consent in accordance with the Declaration of Helsinki.

### Statistical analysis

Descriptive data collected at baseline and after 5 years are presented as mean (SD) or as a percentage. For those patients having a single data point, i.e., ESR, CRP, TJC/SJC, VAS scales and HAQ, missing from the RA-register data, the previous value was used to impute data once for each inflammatory variable assessed, up to 24 months. The proportions of imputations for each variable and registration were 3.6 % from 0 to 6 months, however that figure increased somewhat up to 24 months and is estimated to be at most 16 %. Disease activity, DAS28, was calculated according to the trapezoid model to evaluate the total burden of disease activity over time, referred to as the area under the curve (AUC) of DAS28, at 6, 12, and 24 months after inclusion into the study. Simple and multiple logistic regression analysis was used to evaluate the association between prognostic risk factors and the outcomes. Variables for multiple modeling were chosen based on clinical assumptions and with respect to the results of simple regression analyses (*p* <0.05). In multiple models the influence of age, sex, disease activity (baseline ESR), disease severity (development of Ex-RA), having a smoking history at baseline, and ever using corticosteroids, was examined on different outcome variables; co-morbidity overall, and composite outcome variables such as pulmonary co-morbidity (RA-associated and obstructive lung disease) and endocrine co-morbidity (thyroid disease, osteoporosis, diabetes, hyperparathyroidism). ESR at baseline was chosen as a measure of disease activity because data on baseline ESR was available for more than 95 % of patients compared with AUC for DAS28 at 24 months, for which we had fewer data. All *p* values are two-sided, and *p* values ≤0.05 were considered statistically significant. All calculations were performed using the IBM SPSS Statistics 21.0 program (SPSS, Chicago, IL, USA).

## Results

### Demographic data at baseline (T0) and after 5 years (T5)

The mean age at disease onset was 55.6 years (range 18–89 years). Of the 950 patients included at baseline (T0), 649 (68.3 %) were female and 301 (31.7 %) male. The mean duration (SD) from the first symptom of rheumatoid disease to inclusion into the register was 6.7 (3.5) months (Table 1).

**Table 1:** Descriptive data at baseline (T0) for 950 patients and at 5-year follow up (T5) in 726 of the patients diagnosed with early rheumatoid arthritis

Characteristics	Values
Age at onset of symptoms, years	55.6 (14.4)
Female/male, T0	649/301 (68.3/31.7)
Duration of symptoms at T0, months	6.7 (3.5)
RF+	75.3
ANA+	22.0
ACPA+	69.0
HLA-SE+	58.0
Disease activity and severity at T0	
ESR, mm/h	30.1 (23.2)
CRP, mg/L	20.7 (24.3)
HAQ	0.86 (0.6)
Tender joint count	6.4 (5.7)
Swollen joint count	7.0 (5.2)
VAS pain, mm	43.4 (25.7)
VAS global, mm	44.4 (25.7)
AUC DAS28, 6 months	25.1 (7.2)
AUC DAS28, 12 months	46.2 (13.4)
AUC DAS28, 24 months	86.5 (25.6)
Ex-RA ≤ T5	4.9
Nodules ≤ T5	16.4
Smoking ever, T0	65.8
Treatments	
DMARDs within 3 months after T0	90.1
DMARDs ever ≤ T5	97.8
Methotrexate ever ≤ T5	86.9
Biological treatment ever ≤ T5	16.5
Time without DMARDs from symptom onset, months	6.8 (4.9)
Time without DMARDs T0–T5, months	8.4 (15.7)
NSAIDs ever ≤ T5	84.0
Cox-2 inhibitors ever ≤ T5	26.5
Corticosteroids T0–T5, months	22.2 (23.8)
Corticosteroids ever ≤ T5	71.0

Mean(SD), number or percent. *RF* rheumatoid factor, *ANA* anti-nuclear antibody, *ACPA* anti-citrullinated protein/peptide antibody (analyzed as anti-CCP2), *HLE-SE* human leucocyte antigen-shared epitope, *ESR* erythrocyte sedimentation rate, *CRP* C-reactive protein, *HAQ* health assessment questionnaire, *AUC* area under curve, *DAS28* disease activity score in 28 joints, *Ex-RA* extra articular manifestations of RA, *DMARDs* disease modifying anti-rheumatic drugs, *NSAIDs* non-steroid anti-inflammatory drugs, *Cox-2* cyclo oxygenase 2inhibitors

In all, 53.2 % of patients had one or more co-morbidity at the onset of RA. The most common co-morbidities at inclusion were hypertension (27.3 %), obstructive pulmonary disease (asthma and/or COPD) (13.9 %), diabetes (8.0 %), hypothyroidism (6.3 %) and malignancy (5.0 %). After 5 years 41.0 %, had developed a minimum of one new co-morbidity, of whom 27.8 % had one co-morbidity, 9.1 %, had two, 3.4 % had three, and 0.7 % had four co-morbidities. The commonest new co-morbidities were hypertension (15.1 %), malignancy (7.6 %), stroke/TIA (5.1 %), myocardial infarction (MI) (4.3 %) and osteoporosis (3.7 %).

Among the patients with obstructive pulmonary disease at inclusion 39 patients (4.1 %) had COPD and 106 (11.2 %) had asthma. Of these, 13 patients had both COPD and asthma. After 5 years, 13 patients (1.8 %) had developed COPD and 4 (0.6 %) asthma.

During follow up, 40 patients (4.9 %) suffered an extra-articular manifestation. Twelve patients already had Ex-RA at T0. All of these manifestations constituted RA-associated lung disease. At T5, 28 patients developed a new Ex-RA, including 20 patients with RA-associated lung disease. Thus, of the 40 patients with Ex-RA, at follow up as many as 32 were identified as having RA-associated lung disease. For analysis of predictors for new lung disease, a composite variable was created comprising obstructive lung disease COPD/asthma and RA-associated lung disease (n = 37 in all). The composite variable for a new endocrine disease during 5 years comprised 75 patients (10.5 %), including patients with thyroid disease (2.4 %), osteoporosis (3.7 %), DM (3.3 %), and hyperparathyroidism (0.6 %) (Table 2).

**Table 2:** Co-morbidity at baseline and 5 years in patients with early rheumatoid arthritis

	Baseline (n =950)	New co-morbidity during 5 years of disease (n = 726)
	%	%
Co-morbidity overall^a^	53.2	41.0
Hypertension	27.3	15.1
Asthma/COPD	13.9 (11.2/4.1)	2.4 (0.6/1.8)
Any endocrine disease	19.2	10.5
Diabetes mellitus	8.0	3.3
Hypothyroidism	6.3	1.9
Thyroid disease^b^	10.4	2.4
Osteoporosis	1.4	3.7
Hyperparathyroidism	0.6	0.6
Malignancy	5.0	7.6
Myocardial infarction	4.5	4.3
Stroke/TIA	3.9	5.1

Values are percent for all. ^a^Co-morbidity defined according to Charlson [40]. ^b^Defined as hypothyroidism, hyperthyroid disease and goiter. *COPD* chronic obstructive lung disease, *TIA* transient ischemic attack

### Predictors of a new co-morbidity after five years: simple logistic regression models

In simple regression analyses, co-variates associated with a new co-morbidity after 5 years were age at inclusion, presence of co-morbidity at baseline, ESR at baseline, corticosteroid therapy (*p* <0.001 for all), Ex-RA (*p* <0.01), AUC DAS28 (24 months) (*p* <0.05), ever smoking (*p* = 0.058) and male gender (*p* <0.01). The covariate associated with reduced risk of a new co-morbidity was treatment with a biological agent (*p* <0.05) (Table 3).

**Table 3:** Covariates associated with increased or reduced risk of a new co-morbidity overall during the first five years in simple logistic regression analysis

Covariates	Odds ratio	CI 95 %	*P* value
ESR (T0), mm/h	1.015	1.008, 1.022	<0.001
AUC DAS28 (24 months)	1.008	1.000, 1.016	<0.05
Ex-RA, yes/no	3.247	1.604, 6.574	<0.01
Corticosteroids ever ≤ T5	2.087	1.468, 2.969	<0.001
Biological treatment ever ≤ T5	0.599	0.389, 0.922	<0.05
Smoking, ever (T0)	1.373	0.990, 1.905	0.058
Age at disease onset, years	1.068	1.054, 1.083	<0.001
Sex male/female	1.539	1.118, 2.119	<0.01
Body mass index (T0)	1.034	0.990, 1.080	0.129
Co-morbidity at T0, number	1.459	1.248, 1.704	<0.001

*ESR* erythrocyte sedimentation rate, *T0* baseline, *AUC* area under the curve, *DAS28* disease activity score in 28 joints, *Ex-RA* extra-articular disease, *T5* five years

### Predictors of a new co-morbidity after five years: multiple logistic regression models

In multiple regression analyses, ESR at baseline was significantly associated with a new co-morbidity after 5 years in a model adjusted for age, sex, smoking habits and corticosteroid therapy (Table 4). In a similar model substituting ESR with Ex-RA, Ex-RA (OR 2.245 CI 1.036, 4.868); *p* <0.05) was associated with a new co-morbidity. In a model evaluating predictors for new lung co-morbidity, including RA-associated lung disease, and smoking habit - but not ESR at baseline - was significantly associated with the outcome (Table 5). For variables associated with a new endocrine co-morbidity as one group, ESR at baseline approached significance (*p* = 0.10) in a model adjusted for age, sex, smoking habits and corticosteroid treatment (Table 6). Adjustments for presence of co-morbidities at baseline had no impact on any of the presented models.

**Table 4:** Covariates associated with a new co-morbidity overall during the first five years

Co-variates	Odds ratio	CI 95 %	*P* value
ESR. T0, mm/h	1.008	1.001, 1.016	0.036
Corticosteroids ever ≤ T5	1.760	1.168, 2.654	<0.01
Smoking ever, T0	1.302	0.877, 1.933	0.190
Age at disease onset, years	1.072	1.055, 1.090	<0.001
Sex female/male	1.058	0.720, 1.554	0.775

Multiple logistic regression analysis (n = 623). *ESR* erythrocyte sedimentation rate, *T0* baseline, *T5* five years after baseline

**Table 5:** Covariates associated with a new lung co-morbidity (obstructive lung disease, rheumatoid arthritis-associated lung disease) during the first five years

Covariates	Odds ratio	CI 95 %	*P* value
ESR, T0, mm/h	1.002	0.988, 1.017	0.760
Smoking ever, T0	6.479	1.925, 21.800	<0.01
Age at disease onset, years	1.043	1.010, 1.078	<0.05
Sex female/male	1.663	0.765, 3.618	0.199

Multiple logistic regression analysis (n = 637). *ESR* erythrocyte sedimentation rate, *T0* baseline, *T5* five years after baseline

**Table 6:** Covariates associated with new endocrine disease (thyroid diseases, osteoporosis, diabetes, hyperparathyroidism) during the first five years

Co-variates	Odds ratio	CI 95 %	*P* value
ESR (T0), mmHg	1.009	0.998, 1.020	0.101
Corticosteroids ever ≤ T5	1.542	0.792, 3.002	0.202
Smoking ever (T0)	0.761	0.435, 1.332	0.339
Age at disease onset, years	1.052	1.028, 1.077	<0.001
Sex f/m	4.552	2.076, 9.980	<0.001

Multiple logistic regression analysis (n = 625). *ESR* erythrocyte sedimentation rate, *T0* baseline, *T5* five years after baseline

## Discussion

The objectives of the present study were to investigate, first, the presence of co-morbidities in patients with early RA and the spectrum of new co-morbidities developing during the first five years following diagnosis, and second, the impact of inflammation on the development of a new co-morbidity during the first 5 years following diagnosis.

The presence of co-existing disease was common within this cohort of patients with early RA. In all, 53.2 % of the patients had at least one medical condition in addition to RA, of whom 23 % had more than one co-existing disease at RA onset. These figures are close to those reported for cohorts of patients with early RA from North America (58 % [8]), the Netherlands (66 %, [43]), Great Britain (31.6 % [10]) and Southern Sweden (43 % [44]). In contrast, a recent study reported a lower rate of co-morbidity with a baseline prevalence of 20 % for all co-morbidities in patients with early RA [11]. In the present study the burden of co-existing disease increased during the 5-year follow up period and 41.0 % developed at least one new co-morbidity over the first 5 years of disease. Furthermore, these figures are consistent with other reports of an increasing burden of illness over time [8, 10, 11, 44]. However, it is difficult to compare the overall disease burden among the various studies since there is no common definition of co-morbidity. We used the Charlson co-morbidity index to define the co-morbidity groups. This index was developed to predict one-year mortality in a large cohort of patients and comprises a weighting of diagnosis for mortality risk. The focus of the present study was, however, the role of inflammation in the context of the development of co-morbidity. Thus, we only used this instrument for the purpose of defining co-morbidities.

In this cohort of 950 patients with early RA, the mean age at onset was 55.6 years and 27.3 % were receiving treatment for hypertension at inclusion, whilst an additional 15.1 % developed hypertension during the first 5 years of disease. This figure is higher than expected. The prevalence of hypertension requiring treatment in Swedes aged 60 years was calculated to be 10 % in 2003 [45]. The occurrence of chronic pulmonary disease, in this context of COPD and/or asthma, at the onset of RA was close to 14 %, of whom most had been diagnosed with asthma. Furthermore, approximately 85 % of the patients with chronic pulmonary disease (COPD and/or asthma) were diagnosed before the onset of RA, which is consistent with previous reports [9, 43]. Previous studies have shown RA patients to have a higher than expected risk of COPD [46] and an increased risk of asthma [47] compared with the general population but the reported data are conflicting, especially for asthma [48]. Regarding Ex-RA, almost 5 % of the patient cohort developed such a complication during the 5-year follow up period with RA-associated lung disease being the most common comprising 4 % of the total 5 %. More than a third already had an RA-associated lung disease at the onset of RA; a finding that is in keeping with Koduri et al. [20]. When considering the emerging data suggesting the mucosal surfaces, i.e., the lung, to be the potential site for an initial immune dysregulation and autoantibody generation in the early development of RA [49], the relatively high proportion of patients with lung disease involvement at baseline is of great interest. When we used a multiple model to evaluate predictors of developing a new lung disease, disease activity, measured as ESR, had no impact, whereas having ever being a smoker was independently associated with the outcome.

In univariate analysis cumulative inflammation over time (i.e., AUC DAS28 after 24 months) was associated with a new co-morbidity. Furthermore, ESR at inclusion was independently associated with a new co-morbidity during the first 5 years in a multiple model adjusted for age, sex, use of corticosteroids and smoking. Also, a more serious disease, measured by the development of Ex-RA during the follow up period, was associated with a new co-morbidity over time. However, others were unable to demonstrate that co-morbidity is related to disease activity [10, 44].

In all, 6.3 % of the patients had hypothyroidism at the time of RA onset, a finding that is consistent with that of Raterman et al., who reported an increased risk of hypothyroidism in female RA patients compared with controls [50], but in contrast with a recent study reporting no increase of hypothyroid diseases in patients with RA compared to those without [51]. However, both of these studies reported that hypothyroid disease in RA patients was associated with increased risk of CVD and Raterman suggested that RA patients should be screened for hypothyroidism [50]. In an epidemiological study, thyroxine substitution was suggested to be associated with an increased risk of developing RA [52]. In the present cohort, there were strong gender differences with an overall four-fold increased risk of endocrine diseases in female patients. The role of inflammation has been questioned in the context of endocrine disease, [51] however, we did find indications of increased risk; a new endocrine co-morbidity, tended to be associated with higher baseline ESR, and when adjusted for age, sex, smoking and corticosteroid treatment. During the 5-year follow up period, 7.6 % (n = 54) developed a new malignancy, the three most frequent forms being prostate cancer (n = 13), gastrointestinal cancer (n = 10) and lung cancer (n = 10). All of the patients who developed lung cancer had a history of smoking except one patient for whom data on smoking status was lacking. Smoking is a well-known risk factor for both RA and lung cancer, however, systemic inflammation has also been reported to be associated with lung cancer [26]. Three patients had developed lymphoma at follow up. Higher inflammatory activity has been shown to be a major risk factor for lymphoma in RA patients and the chronic inflammatory state associated with RA may be the explanation, however, treatment with DMARDs did not appear to be a risk factor in that study [27].

Concerning cardiovascular co-morbidity, 4.3 % of patients had a new MI and 5.1 % a stroke/TIA during the follow up period. We analyzed co-morbidity thoroughly in a previous prospective study [41] and found inflammatory activity to be harmful in terms of new cardiovascular events; a conclusion that is in keeping with several other cross-sectional and retrospective studies [31–33], but at variance with a smaller study from southern Sweden [44]. We also found that inflammation potentiated the effect of traditional cardiovascular risk factors [41].

The main strengths of the present study are the patient group comprising a large regional cohort, and the prospective design. The study involves few physicians at each rheumatology center in northern Sweden. Essentially, all patients with newly diagnosed RA in Sweden are referred to a specialist. Thus, the results for the present cohort can be regarded as applicable to all patients with early RA. Furthermore, repeated measurement of the variables associated with inflammation made it possible to take the variability in disease activity into account. Conversely, a limitation is the observational nature of this study with a risk of confounding by indication when evaluating the efficacy of pharmacological treatment such as corticosteroids and biologic agents. In the evaluation of the total burden of disease activity over time, we have accurate data only up to 24 months. Another limitation is the relatively small number in each group after any stratification of the data, which made it difficult to perform sub-group analyses. As we have classified our patients according to Charlson [40], we have no reliable data on, for example, mental illnesses such as depression. The most common disease group of myocardial and vascular diseases, including hypertension, was the focus of a previous study and has been presented in detail [41].

## Conclusion

In conclusion, co-morbidity was found to be common in patients with recent onset of RA, and considerable new co-morbidity developed during the first 5 years of disease. We were also able to show inflammatory activity, both at disease onset and accumulated over time, to be associated with a new co-morbidity during the follow up period. Furthermore, there was a tendency for inflammation to predict endocrine disease. Of interest was that inflammation did not predict a new lung co-morbidity, but smoking was a strong predictor.

To summarize, in addition to the index disease, patients with RA appear to have several other medical conditions at disease onset that add to their disease burden. Thus, in everyday clinical practice, the patient's disease activity should be treated not only to prevent any destruction of joints but also with regard to their specific co-morbid disease(s). The need to take preventive action through lifestyle changes, in particular in relation to smoking, should be emphasized in the modern care of rheumatics.

## Abbreviations

ACPA: antibodies against cyclic citrullinated peptides/proteins
ANA: anti-nuclear antibodies
AUC: area under the curve
BMI: body mass index
CABG: coronary artery bypass grafting
CI: confidence interval
COPD: chronic obstructive pulmonary disease
CV: cardiovascular
CVD: cardiovascular disease
CVE: cardiovascular event
COX-2: cyclo-oxygenase-2
CRP: C-reactive protein
DAS28: disease activity score in 28 joints
DM: diabetes mellitus
DMARD: disease-modifying anti-rheumatic drug
DVT: deep vein thrombosis
ELISA: enzyme-linked immunoassay
ESR: erythrocyte sedimentation rate
Ex-RA: extra-articular disease
HAQ: Health Assessment Questionnaire
HLA: human leucocyte antigen
HT: hypertension
MI: myocardial infarction
NSAID: non-steroidal anti-inflammatory drug
OR: odds ratio
PE: pulmonary embolism
PTPN22: protein tyrosine phosphatase non-receptor type 22
RA: rheumatoid arthritis
RF: rheumatoid factor
SD: standard deviation
SE: shared epitope
SJC: swollen joint count
TIA: transient ischemic attack
TJC: tender joint count
T0: baseline
T5: 5-year patient follow up
VAS: visual analog scale

## References

[1] Turesson C, Jacobsson LT (2004). Epidemiology of extra-articular manifestations in rheumatoid arthritis. Scand J Rheumatol..

[2] Young A, Koduri G (2007). Extra-articular manifestations and complications of rheumatoid arthritis. Best Pract Res Clin Rheumatol..

[3] Wallberg-Jonsson S, Ohman ML, Dahlqvist SR (1997). Cardiovascular morbidity and mortality in patients with seropositive rheumatoid arthritis in Northern Sweden. J Rheumatol..

[4] Sokka T, Abelson B, Pincus T (2008). Mortality in rheumatoid arthritis: 2008 update. Clin Exp Rheumatol..

[5] Wolfe F, Mitchell DM, Sibley JT, Fries JF, Bloch DA, Williams CA (1994). The mortality of rheumatoid arthritis. Arthritis Rheum..

[6] Gonzalez A, Maradit Kremers H, Crowson CS, Nicola PJ, Davis JM, Therneau TM (2007). The widening mortality gap between rheumatoid arthritis patients and the general population. Arthritis Rheum..

[7] Gabriel SE, Michaud K (2009). Epidemiological studies in incidence, prevalence, mortality, and comorbidity of the rheumatic diseases. Arthritis Res Ther..

[8] Gabriel SE, Crowson CS, O'Fallon WM (1999). Comorbidity in arthritis. J Rheumatol..

[9] Hyrich K, Symmons D, Watson K, Silman A, Consortium BCC, British Society for Rheumatology Biologics R (2006). Baseline comorbidity levels in biologic and standard DMARD treated patients with rheumatoid arthritis: results from a national patient register. Ann Rheum Dis..

[10] Norton S, Koduri G, Nikiphorou E, Dixey J, Williams P, Young A (2013). A study of baseline prevalence and cumulative incidence of comorbidity and extra-articular manifestations in RA and their impact on outcome. Rheumatology (Oxford).

[11] Tiippana-Kinnunen T, Kautiainen H, Paimela L, Leirisalo-Repo M (2013). Co-morbidities in Finnish patients with rheumatoid arthritis: 15-year follow-up. Scand J Rheumatol..

[12] Michaud K, Wolfe F (2007). Comorbidities in rheumatoid arthritis. Best Pract Res Clin Rheumatol..

[13] Gullick NJ, Scott DL (2011). Co-morbidities in established rheumatoid arthritis. Best Pract Res Clin Rheumatol..

[14] del Rincon ID, Williams K, Stern MP, Freeman GL, Escalante A (2001). High incidence of cardiovascular events in a rheumatoid arthritis cohort not explained by traditional cardiac risk factors. Arthritis Rheum..

[15] Kremers HM, Crowson CS, Therneau TM, Roger VL, Gabriel SE (2008). High ten-year risk of cardiovascular disease in newly diagnosed rheumatoid arthritis patients: a population-based cohort study. Arthritis Rheum..

[16] Sodergren A, Stegmayr B, Lundberg V, Ohman ML, Wallberg-Jonsson S (2007). Increased incidence of and impaired prognosis after acute myocardial infarction among patients with seropositive rheumatoid arthritis. Ann Rheum Dis..

[17] Kerola AM, Kauppi MJ, Kerola T, Nieminen TV (2012). How early in the course of rheumatoid arthritis does the excess cardiovascular risk appear?. Ann Rheum Dis..

[18] Dawson JK, Fewins HE, Desmond J, Lynch MP, Graham DR (2001). Fibrosing alveolitis in patients with rheumatoid arthritis as assessed by high resolution computed tomography, chest radiography, and pulmonary function tests. Thorax..

[19] Bongartz T, Nannini C, Medina-Velasquez YF, Achenbach SJ, Crowson CS, Ryu JH (2010). Incidence and mortality of interstitial lung disease in rheumatoid arthritis: a population-based study. Arthritis Rheum..

[20] Koduri G, Norton S, Young A, Cox N, Davies P, Devlin J (2010). Interstitial lung disease has a poor prognosis in rheumatoid arthritis: results from an inception cohort. Rheumatology (Oxford).

[21] Doran MF, Crowson CS, Pond GR, O'Fallon WM, Gabriel SE (2002). Frequency of infection in patients with rheumatoid arthritis compared with controls: a population-based study. Arthritis Rheum..

[22] Listing J, Gerhold K, Zink A (2013). The risk of infections associated with rheumatoid arthritis, with its comorbidity and treatment. Rheumatology (Oxford).

[23] Ebert EC, Hagspiel KD (2011). Gastrointestinal and hepatic manifestations of rheumatoid arthritis. Dig Dis Sci..

[24] Haugeberg G, Green MJ, Quinn MA, Marzo-Ortega H, Proudman S, Karim Z (2006). Hand bone loss in early undifferentiated arthritis: evaluating bone mineral density loss before the development of rheumatoid arthritis. Ann Rheum Dis..

[25] van Staa TP, Geusens P, Bijlsma JW, Leufkens HG, Cooper C (2006). Clinical assessment of the long-term risk of fracture in patients with rheumatoid arthritis. Arthritis Rheum..

[26] Smitten AL, Simon TA, Hochberg MC, Suissa S (2008). A meta-analysis of the incidence of malignancy in adult patients with rheumatoid arthritis. Arthritis Res Ther..

[27] Baecklund E, Iliadou A, Askling J, Ekbom A, Backlin C, Granath F (2006). Association of chronic inflammation, not its treatment, with increased lymphoma risk in rheumatoid arthritis. Arthritis Rheum..

[28] Askling J, Fored CM, Brandt L, Baecklund E, Bertilsson L, Feltelius N (2005). Risks of solid cancers in patients with rheumatoid arthritis and after treatment with tumour necrosis factor antagonists. Ann Rheum Dis..

[29] Maradit-Kremers H, Crowson CS, Nicola PJ, Ballman KV, Roger VL, Jacobsen SJ (2005). Increased unrecognized coronary heart disease and sudden deaths in rheumatoid arthritis: a population-based cohort study. Arthritis Rheum..

[30] Avina-Zubieta JA, Choi HK, Sadatsafavi M, Etminan M, Esdaile JM, Lacaille D (2008). Risk of cardiovascular mortality in patients with rheumatoid arthritis: a meta-analysis of observational studies. Arthritis Rheum..

[31] Wallberg-Jonsson S, Johansson H, Ohman ML, Rantapaa-Dahlqvist S (1999). Extent of inflammation predicts cardiovascular disease and overall mortality in seropositive rheumatoid arthritis. A retrospective cohort study from disease onset. J Rheumatol.

[32] Maradit-Kremers H, Nicola PJ, Crowson CS, Ballman KV, Gabriel SE (2005). Cardiovascular death in rheumatoid arthritis: a population-based study. Arthritis Rheum..

[33] del Rincon I, Freeman GL, Haas RW, O'Leary DH, Escalante A (2005). Relative contribution of cardiovascular risk factors and rheumatoid arthritis clinical manifestations to atherosclerosis. Arthritis Rheum..

[34] Välkommen till Svenska Reumatologi Register. www.srq.nu.

[35] Arnett FC, Edworthy SM, Bloch DA, McShane DJ, Fries JF, Cooper NS (1988). The American Rheumatism Association 1987 revised criteria for the classification of rheumatoid arthritis. Arthritis Rheum..

[36] Ekdahl C, Eberhardt K, Andersson SI, Svensson B (1988). Assessing disability in patients with rheumatoid arthritis. Use of a Swedish version of the Stanford Health Assessment Questionnaire. Scand J Rheumatol.

[37] Prevoo ML, van't Hof MA, Kuper HH, van Leeuwen MA, van de Putte LB, van Riel PL (1995). Modified disease activity scores that include twenty-eight-joint counts. Development and validation in a prospective longitudinal study of patients with rheumatoid arthritis. Arthritis Rheum.

[38] Berglin E, Padyukov L, Sundin U, Hallmans G, Stenlund H, Van Venrooij WJ (2004). A combination of autoantibodies to cyclic citrullinated peptide (CCP) and HLA-DRB1 locus antigens is strongly associated with future onset of rheumatoid arthritis. Arthritis Res Ther..

[39] Kokkonen H, Johansson M, Innala L, Jidell E, Rantapaa-Dahlqvist S (2007). The PTPN22 1858C/T polymorphism is associated with anti-cyclic citrullinated peptide antibody-positive early rheumatoid arthritis in northern Sweden. Arthritis Res Ther..

[40] Charlson ME, Pompei P, Ales KL, MacKenzie CR (1987). A new method of classifying prognostic comorbidity in longitudinal studies: development and validation. J Chronic Dis..

[41] Innala L, Moller B, Ljung L, Magnusson S, Smedby T, Sodergren A (2011). Cardiovascular events in early RA are a result of inflammatory burden and traditional risk factors: a five year prospective study. Arthritis Res Ther..

[42] Turesson C, Jacobsson L, Bergstrom U (1999). Extra-articular rheumatoid arthritis: prevalence and mortality. Rheumatology (Oxford).

[43] Kroot EJ, van Gestel AM, Swinkels HL, Albers MM, van de Putte LB, van Riel PL (2001). Chronic comorbidity in patients with early rheumatoid arthritis: a descriptive study. J Rheumatol..

[44] Kapetanovic MC, Lindqvist E, Simonsson M, Geborek P, Saxne T, Eberhardt K (2010). Prevalence and predictive factors of comorbidity in rheumatoid arthritis patients monitored prospectively from disease onset up to 20 years: lack of association between inflammation and cardiovascular disease. Scand J Rheumatol..

[45] evidensbaserad äldrevård*.* http://www.sbu.se/upload/Publikationer/Content0/2/aldrevard_2003/aldrevardfull.html.

[46] Bieber V, Cohen AD, Freud T, Agmon-Levin N, Gertel S, Amital H (2013). Autoimmune smoke and fire–coexisting rheumatoid arthritis and chronic obstructive pulmonary disease: a cross-sectional analysis. Immunol Res..

[47] Shen TC, Lin CL, Wei CC, Tu CY, Li YF (2014). The risk of asthma in rheumatoid arthritis: a population-based cohort study. QJM.

[48] Rudwaleit M, Andermann B, Alten R, Sorensen H, Listing J, Zink A (2002). Atopic disorders in ankylosing spondylitis and rheumatoid arthritis. Ann Rheum Dis..

[49] Demoruelle MK, Deane KD, Holers VM (2014). When and where does inflammation begin in rheumatoid arthritis?. Curr Opin Rheumatol..

[50] Raterman HG, van Halm VP, Voskuyl AE, Simsek S, Dijkmans BA, Nurmohamed MT (2008). Rheumatoid arthritis is associated with a high prevalence of hypothyroidism that amplifies its cardiovascular risk. Ann Rheum Dis..

[51] McCoy SS, Crowson CS, Gabriel SE, Matteson EL (2012). Hypothyroidism as a risk factor for development of cardiovascular disease in patients with rheumatoid arthritis. J Rheumatol..

[52] Bengtsson C, Padyukov L, Kallberg H, Saevarsdottir S (2014). Thyroxin substitution and the risk of developing rheumatoid arthritis; results from the Swedish population-based EIRA study. Ann Rheum Dis.

